# Novel Targeted Therapies for T-Cell Malignancies

**DOI:** 10.3390/cancers14163955

**Published:** 2022-08-16

**Authors:** Melania Tesio

**Affiliations:** UR LIB “Lymphoma Immune Biology”—INSERM U111, Centre International de Recherche en Infectiologie (CIRI), 69007 Lyon, France; melania.tesio@inserm.fr

T-cell malignancies comprise a heterogeneous group of cancers resulting from the clonal expansion of T-cells at different developmental stages. They arise from the transformation of thymic immature progenitor cells, such as T-cell acute lymphoblastic leukemia (T-ALL), or post-thymic mature T-cells, such as T-cell lymphomas. This group is broadly divided into two categories, namely peripheral T-cell lymphoma (PTCL) and cutaneous T-cell lymphomas (CTCL), which is characterized by malignant T-cells infiltrating the skin. While mycosis fungoides (MF) and Sezary syndrome (SS) are the two most common CTCL subtypes, PTCL can be further classified into several different subtypes.

The heterogeneity of T-cell malignancies, which parallels the heterogeneity in the T-cell maturation stage, functions and tissue-specific distribution, reflects prognostic differences. While risk-based therapeutic regimens in T-ALL reached 80% overall survival for pediatric patients, relapse and treatment-related toxicities remain clinical challenges, respectively, in pediatric and adult settings [1]. With the exception of ALK-positive anaplastic large-cell lymphoma (ALCL), most peripheral T-cell lymphomas are aggressive diseases, presenting poor clinical outcomes and high relapse rates following first-line treatments [2].

Despite their clinical-biological heterogeneity, T-cell malignancies share certain genetic lesions such as those leading to the aberrant activation of JAK/STAT, PI3K-mTOR and NF-kB signaling. This provides a good rational for targeting these signal transduction pathways with small molecule inhibitors. JAK/STAT inhibitors represent interesting targeted drugs in SS [3], in JAK-mutated T-ALL (for which the FDA-approved JAK1/2 inhibitor ruxolinitib is under clinical trail (ClinicalTrials.gov Identifier: NCT03613428)) and in certain PTCL subtypes (such as the enteropathy-associated T-cell lymphoma, where the JAK/STAT pathway is the most commonly mutated [4]). Moreover, combined to venetoclax (BCL2-inhibitor), ruxolinitib induced an encouraging response in two JAK3-mutant T-cell prolymphocytic leukemia patients, warranting further explorations of this drug combination for this rare and aggressive leukemic form [5], where JAK3 mutations are the only lesion endowed with a significant negative prognostic impact [6]. Targeting the PI3K-AKT-mTOR pathway represents another potential strategy in distinct T-cell tumors such as relapsed/refractory PTCL and CTCL, where dual PI3Kd/g inhibitors such as duvelisib and tenalisib showed promising results [7], and in T-ALL, where a variety of genetic and non-genetic mechanisms induce an oncogenic activation of this pathway [8,9,10]. Another promising drug is the proteasome inhibitor bortezomib, which precludes NF-kB activation. This pathway is mutated in several T-cell malignancies (such as ANKL, MF, SS and ATLL [11,12] and it is aberrantly activated in T-ALL [13]. Its administration during re-induction chemotherapy induced an encouraging complete remission rate in pediatric relapsed T-ALL patients [14]. In combination therapies, bortezomib is also under clinical investigation for PTCL patients (ClinicalTrials.gov Identifier: NCT02783625).

Significant progress has been also made in targeting disease subtype-specific pathways. An example is the NOTCH1 pathway, which plays a major pathogenetic role in T-ALL. The use of γ-secretase inhibitors, which prevent the proteolytic step leading to NOTCH1 activation, has been rather disappointing due to the excessive gastro-toxicity observed in the clinical setting. This has prompted the development of a number of potential alternatives. These include monoclonal antibodies [15], the PSEN1 inhibitor MK-560, which showed promising results in pre-clinical studies [16], and SERCA inhibitors [17], which prevent NOTCH1 localization to the cell membrane. Another example of subtype-specific druggable mutations is the ALK fusion gene, which defines a specific subset of anaplastic large cell lymphomas (ALCL). The identification of the *ALK* fusion, which leads to the constitutive activation of ALK kinase, has led to the development of ALK inhibitors such as crizotinib, which has been approved by the FDA for the treatment of children and young adult with replaced/refractory ALCL [18].

Epigenetic changes are also frequently found in T-ALL and multiple subtypes of T-cell lymphomas and they mostly result from mutations in genes regulating DNA methylation and histone modifications such as acetylation. So far, a number of histone deacetylase inhibitors (HDACi) have been approved by the FDA for the treatment of relapse/refractory CTCL (i.e., vorinostat and romidepsin) and relapsed or refractory PTCL (i.e., romidepsin and belinostat). However, in the studies which lead to their FDA approval, these drugs only achieved overall responses in around 25–30% of the patients [19,20,21]. While these data call for the investigation of combination therapies, the responsiveness to HDACi is likely linked to a follicular helper phenotype [22], thus indicating a subtype-specific efficacy for these compounds. Among epigenetic drugs, inhibitors of bromodomain-containing proteins (BET inhibitors), such as JQ-1, have also become important in the last few years. In T-ALL, these drugs show promise as pharmacologically targeting super-enhancers [23]. Given that JQ-1 short half life limits its use in vivo, novel BET inhibitors are under development and pre-clinical testing is being carried out in T-ALL and CTCL [24,25]. Targeting DNA methyltransferase provided interesting results in AITL [26] and relapsed ETP-ALL [27]. Moreover, in combination therapy with HDCAi, the demethylating drug 5-azacytidine showed good responses in PTCL [28].

When it comes to immunotherapy, promising results have been obtained in T-cell lymphomas with antibody-based therapies. Bretuximab vedotin, an anti-CD30 antibody conjugated to the microtubule-disrupting agent monomethylauristatin E, has been approved for relapsed/refractory CD30+ systemic ALCL as well as for CD30+ CTCL and primary cutaneous ALCL [29,30]. Mogamulizumab, a monoclonal antibody directed against the CCR4 receptor, has instead been approved for the treatment of refractory MF and SS as well as for ATLL [31]. While a number of other mAbs are in clinical trials for T-cell lymphomas (i.e., PH4102—anti-KIR3DL2; daratumumab—anti-CD38; basiliximab and camidanlumab—anti-CD25; TTI-621—anti-CD47; MEDI-570—anti-ICOS; alemtuzumab anti-CD52; AFM13—bispecific antibody targeting both CD30 and CD16A) [32,33], antibody-based therapies have been less successful in T-ALL, where in adult settings the anti-CD38 antibody isatuximab did not show responses, inducing a premature closure of its clinical testing. In pediatric patients, isatuximab and daratumumab are still being tested (ClinicalTrials.gov Identifier: NCT03384654; EudraCT 2017-003377-34, ClinicalTrials.gov Identifier: NCT03860844). While in some T-cell lymphoma subsets, such as refractory ENKL, targeting the PD-1 checkpoint with the monoclonal antibody pembrolizumab led to promising efficacy with limited toxicities, PD-1 inhibition may represent a double edge word in PTCL where PD-1 could act as a tumor suppressor [34,35,36].

Although chimeric antigen receptor (CAR)-based immunotherapy has been very successful in relapsed/refractory B-cell malignancies, the development of CAR T-cells against T-cell malignancies faces a number of challenges, such as minimizing fratricidal effects among CAR T-cells [37]. In this regard, promising strategies consist of targeting the tumor-specific TCR constant region, using allogeneic CAR T-cells, eliminating CAR T-cell targets in T effector cells through CRISPR technology and developing CAR NK-based strategies. Despite these challenges, encouraging results have been reported, for example with allogeneic anti-CD7 CAR T-cells in relapsed/refractory T-ALL [38] or the use of anti CD30 CAR cells in ALK+ ALCL [39].

To conclude, in the past few years, the extensive molecular, genetic and epigenetic characterization of T-cell malignancies has contributed to our understanding of their heterogeneity, providing new research directions and potential therapeutic targets. Hence, the development of novel targeted drugs, combined with chemotherapy or with each other, has already produced encouraging responses in relapsed/refractory patients. Implementing CAR-based technologies and providing novel understandings of the biology of distinct T-cell tumors, their cell of origin and their interactions with the microenvironment will help to further refine risk-based classifications, discover novel targets and identify novel biomarkers to predict clinical responses. This will be an essential step towards personalized medicine.
